# Sensory Evaluation of E-Liquid Flavors by Smelling and Vaping Yields Similar Results

**DOI:** 10.1093/ntr/ntz155

**Published:** 2019-08-22

**Authors:** Erna J Z Krüsemann, Franziska M Wenng, Jeroen L A Pennings, Kees de Graaf, Reinskje Talhout, Sanne Boesveldt

**Affiliations:** 1 Centre for Health Protection, National Institute for Public Health and the Environment (RIVM), Bilthoven, The Netherlands; 2 Division of Human Nutrition and Health, Wageningen University, Wageningen, The Netherlands

## Abstract

**Introduction:**

Sensory research on e-liquid flavors can be performed by means of smelling and vaping. However, data comparing smelling versus vaping e-liquid flavors are lacking. This study aims to investigate if smelling could be an alternative to vaping experiments by determining the correlation for hedonic flavor assessment between orthonasal smelling and vaping of e-liquids, for smokers and nonsmokers.

**Methods:**

Twenty-four young adult smokers (mean age 24.8 ± 9.3) and 24 nonsmokers (mean age 24.9 ± 7.7) smelled and vaped 25 e-liquids in various flavors. Participants rated liking, intensity, familiarity, and irritation on a 100-mm Visual Analog Scale. Pearson correlations within and between smelling and vaping were calculated. Differences between user groups were calculated using *t* tests.

**Results:**

Correlation coefficients between smelling and vaping based on mean group ratings were 0.84 for liking, 0.82 for intensity, 0.84 for familiarity, and 0.73 for irritation. Means of the within-subjects correlation coefficients were, respectively, 0.51, 0.37, 0.47, and 0.25. Correlations between smelling and vaping varied across individuals (ranging from −0.27 to 0.87) and flavors (−0.33 to 0.81). Correlations and mean liking ratings did not differ between smokers and nonsmokers.

**Conclusions:**

The strong group-level correlations between orthonasal smelling and vaping e-liquid flavors justify the use of smelling instead of vaping in future research. For example, smelling could be used to investigate differences in e-liquid flavor liking between (potential) user groups such as nicotine-naïve adolescents. The more modest within-subject correlations and variation across individuals and flavors merit caution in using smelling instead of vaping in other types of experiments.

**Implications:**

This study supports the use of orthonasal smelling (instead of vaping) e-liquids to measure hedonic flavor perception in some studies where vaping would be inappropriate or not feasible. Examples of research situations where smelling e-liquids may be sufficient are (1) investigating nicotine-naïve individuals (ie, nonusers), (2) investigating individuals under legal age for e-cigarette use (ie, youth and adolescents), (3) investigating brain responses to exposure of e-liquid flavors using functional magnetic resonance imaging or electroencephalogram, and (4) comparing hedonic flavor assessment between adolescent nonusers and current smokers to provide support for future regulations on e-liquid flavors.

## Introduction

The use of electronic cigarettes (e-cigarettes) has become increasingly popular over the past years.^1,2^ Literature describes the variety of flavors being an important reason for e-cigarette use.^3,4^ Not surprisingly, the number and variety of flavors on the e-cigarette market has exploded,^5^ for example, up to 245 unique flavors in the Netherlands in 2017 (A. Havermans, et al., unpublished data, 2019). While most e-cigarette users are concurrent or former smokers,^6–8^ the availability of appealing flavors may also stimulate e-cigarette use among nonsmokers and adolescents.^9–13^

As e-cigarettes are less harmful than cigarettes,^14–18^ smokers’ health may benefit from using e-cigarettes compared to smoking combustible tobacco. However, as e-cigarettes are not safe, use among current nonusers and adolescents should be prevented.^14,19^ Research showed that flavor use and preferences may differ between user groups.^8,9,20–22^ Thus, e-liquid flavors could be regulated in order to maximize public health benefits, and for this, research on flavor preferences among different user groups is needed.

Flavor perception is a combined sensation of olfactory stimuli (smell), gustatory stimuli (taste), and chemesthesis (touch).^23,24^ Examples of chemesthetic sensations in the mouth are the burn of capsaicinoids in chili peppers and the cooling of menthol.^25^ Sensory flavor research can be conducted by means of smelling and tasting, that is, smelling and vaping when investigating e-liquid flavors. Smelling and vaping reflect two different ways of olfactory assessment: orthonasally, where ambient odors enter via the nose when we sniff, and retronasally, caused by the airflow from the back of the mouth and throat to the nose when we eat and swallow (similar to vaping). So far, sensory research on e-liquid perception is limited to a few vaping experiments.^26–28^ Whereas orthonasal smelling experiments only focus on the olfactory component of flavor perception, vaping evokes olfactory, gustatory, as well as chemesthetic sensations. However, although reflecting real consumer behavior, vaping experiments are associated with two important ethical restrictions regarding the study population. That is, participants are required to be over 18 years old, and, when investigating nicotine-containing e-liquids, should be experienced vapers or smokers because of the addictive effect of nicotine.^29^ These restrictions do not apply to smelling experiments, which thus provide the opportunity to investigate adolescents and nonusers. In addition, experiments based on smelling e-liquid flavors are faster and less expensive than vaping experiments, because they do not require the use of e-cigarettes. While orthonasal smelling experiments are a potential alternative approach for sensory research on e-liquids, sensory comparability of smelling and vaping data is yet unknown.

Previous research on food and beverages finds comparable results between orthonasal and retronasal perception. For example, studies on wine and Pisco spirits found comparable ratings between orthonasal (sniffing) and retronasal olfaction (sipping and swallow) in terms of descriptive profiling using trained panelists.^30,31^ Furthermore, although neural responses seem to differ, it was shown that pleasantness ratings were comparable between orthonasal and retronasal presentation of chocolate odor.^32^ Another study found that liking, sweetness, and intensity of e-liquid flavors are primarily driven by the e-liquids’ volatile compounds, indicating that e-liquid flavor perception more strongly depends on (retronasal) olfaction than on taste.^33^ In line with this, we hypothesize that hedonic assessment of e-liquids by means of orthonasal smelling and vaping is comparable, and, thus, that smelling experiments could be used to replace vaping experiments.

To test this hypothesis, this study aims to investigate if hedonic evaluation of e-liquid flavors by orthonasal smelling is correlated with (retronasal) vaping ratings. In addition, the correlation between smelling and vaping will be determined for intensity, familiarity, and irritation, as these factors are known to influence liking.^28,34,35^ As smokers are used to inhalation, flavor perception through vaping may differ between smokers and nonsmokers. Therefore, we also investigate if there are differences between smokers and nonsmokers.

## Methods

### Participants

Participants were recruited from Wageningen and surroundings (The Netherlands) by E-mail, social media, flyers, and word-of-mouth. Twenty-four smokers (50% female; mean age = 24.8 ± 9.3, range 18–54 years old) and 24 nonsmokers (50% female; mean age = 24.9 ± 7.7, range 20–55 years old) were included. Smokers reported to smoke more than 1 cigarette/day on average (mean = 10.2 ± 6.5 cigarettes/day) and not only in the weekends. Nonsmokers were required to have never smoked or have quit smoking for more than 12 months. Panel characteristics are shown in Supplementary Table S1. Sample size (*n* = 24 per group, accounting for potential dropout) was determined using a statistical algorithm with 1000 random samplings of a subset of the study population from preliminary smelling experiments (data not shown), and aimed at identifying a correlation coefficient of at least 0.25–0.45 (based on a correlation between liking and familiarity in the preliminary smelling experiments, as well as on correlations between liking and sweetness, coolness, harshness, and bitterness in previous literature^28^), with more than 95% power and significant at *p* < .01.

Participants were screened using a self-report questionnaire to: be between 18 and 55 years of age; be healthy; never have used an e-cigarette before; and have a good proficiency of the Dutch language. In addition, participants had to have normal olfactory function according to the Sniffin’ Sticks identification test.^36^ Exclusion criteria were: pregnancy or lactating; allergies for any of the product flavors under investigation in this study; employment at the Division of Human Nutrition and Health of Wageningen University; and participation in other medical-scientific research.

Participants who completed the study received a financial compensation; participants who did not pass the screening test received a gift voucher. All participants provided written informed consent at their first visit. The study was approved by the Medical Ethical Committee of Wageningen University (NL65748.081.18).

### Experimental Procedure

Eligible participants were invited for a screening session to determine their olfactory functioning. If they passed the olfactory test (≥12 correct answers out of 16),^36^ they were familiarized with the Visual Analog Scale and the type of e-cigarette used in this study, by taking a maximum of five puffs and rating liking (*how much do you like this flavor?*). The e-cigarette contained a nicotine-free, unflavored e-liquid. Participants decided themselves whether to inhale the vape over their lungs or to directly exhale the vape from their mouth, as long as they did this consistently over all sessions (see Supplementary Table S1).

Test sessions took place in sensory booths, each equipped with a computer, water tap, and tissues. The room was accommodated with a controlled ventilation system of five air changes per hour. Participants were asked to refrain from using scented perfumes on test days, and from smoking, chewing gum, brushing their teeth, and eating or drinking anything apart from water at least 1 hour prior to the test sessions. Two smelling and two vaping sessions were scheduled during which participants assessed 25 e-liquid flavors in total (13 and 12 e-liquids per session) on liking, intensity, familiarity, and irritation. The order of the sessions was counterbalanced across individuals. The time between two sessions was at least 1 week.

Visual analog ratings were collected using EyeQuestion software (Logic8 BV, version 4.11.19). During the test sessions, participants were asked to take one puff (vaping sessions) or to smell the e-liquid sample once (smelling sessions). Between each product, a break of at least 30 seconds was installed to prevent olfactory adaptation. Within each session, the product sequence was randomized. All products were first assessed on liking (*how much do you like this odor/flavor?*). Subsequently, after a 1-minute break, products were assessed on perceived intensity (*how strong do you perceive this odor/flavor?*), familiarity (*how familiar are you with this odor/flavor?*), and irritation (*to what extent does this odor/flavor give you an irritating feeling in your nose/mouth or throat?*). Participants were explicitly asked to only focus on the odor/flavor instead of on overall (vaping) experience. Participants were allowed to rinse their mouth with water between each sample. For hygienic purposes, fresh mouthpieces were used every time a participant assessed a new flavor. No adverse events occurred and all measures and conditions have been reported.

### Materials and Equipment

During the training and test sessions, a 100-mm Visual Analog Scale was used to assess liking, intensity, familiarity, and irritation (left anchor at 10 mm: “Not at all,” right anchor at 90 mm: “Extremely”). Twenty-five commercial e-liquids, from four different brands, were purchased from three online shops. E-liquids contained a base of 70% propylene glycol (PG) and 30% glycerin (VG), and 0 mg/mL nicotine. In order to obtain a high variety of flavors, selection of e-liquid flavors was based on the different categories of our recently published e-liquid flavor wheel^37^: tobacco (American tobacco with hazelnut, Indonesian tobacco, and Oriental tobacco); menthol/mint (mint and peppermint); nuts (hazelnut); spices (fennel and licorice); coffee/tea (coffee and cappuccino); alcohol (piña colada and whiskey); other beverages (cola and energy drink); fruit (strawberry, lemon, banana, and watermelon); dessert (cookie); candy (cotton candy and red candy); other sweets (caramel, chocolate, and vanilla); and unflavored (PG/VG base). For vaping, eGo-type e-cigarettes were used with a battery capacity of 900 mAh, constant voltage, and a coil resistance of 2 Ohm.

### Sample Preparation

For smelling, 10 drops of an e-liquid were put in a 50-mL brown glass vial. For vaping, e-cigarette clearomizers were filled with sufficient e-liquid (with a maximum of 1.6 mL) and covered in tin foil to avoid visual cues. E-cigarettes and vials were labeled with a random three-digit code. Vials and e-cigarettes were cleaned and filled with e-liquid up to 2 days before each test session. The coil was replaced when cleaning the e-cigarettes. Every other week, a new set of e-liquids from the same batch was used. E-liquids were stored within their original package at room temperature. Vials and e-cigarettes filled with e-liquid were stored in the dark at room temperature.

### Data Analysis

Data were analyzed using R statistical software (version 3.5.1). No data were excluded. Results are presented for the whole group and separately for smokers and nonsmokers.

#### Mean Ratings

The mean score and standard error *over all flavors* were calculated for each variable, for both smelling and vaping, separately for smokers and nonsmokers. A constant value of 50 was subtracted in order to center ratings around zero. The effect of assessment type (smelling vs. vaping) and smoking status (smokers vs. nonsmokers) and their interaction were examined using a two-way ANOVA model. The model included the participant as a covariate to allow for repeated (paired) measurements per individual. To correct for multiple testing, Benjamini–Hochberg false discovery rate^38^ adjusted *p* values of less than 5% were considered significant.

Next, *for each flavor separately*, mean scores and standard errors were calculated for each variable. For each flavor, differences between smelling and vaping were compared using paired *t* tests; smokers were compared to nonsmokers for liking using an unpaired *t* test. To correct for multiple testing, Benjamini–Hochberg false discovery rate^38^ adjusted *p* values of less than 5% were considered significant.

#### Correlation Coefficients

Since the correlations between smelling and vaping for each variable depend on data that can be assigned to participants (*n* = 48) as well as flavors (*n* = 25),^39^ correlations were calculated in two different ways. First, for each variable (liking, intensity, familiarity, irritation), Pearson correlation coefficients between smelling and vaping were calculated for the mean ratings per flavor, thus based on 25 pairs of data. This was done for the whole group, and separately for smokers and nonsmokers. To determine if correlations for smokers and nonsmokers were significantly different from zero and from each other (*p* ≤ .01), a Fisher’s *Z*-transformation was applied in order to transform the sampling distribution of the Pearson correlations toward a normal distribution. Transformed correlations were compared using an unpaired *t* test, and *p* values ≤ .01 were considered significant.

Secondly, to allow insight into individual participants, a Pearson correlation was calculated for each individual (ie, within-subjects correlations) for liking, intensity, familiarity, and irritation ratings. These individual correlations were Fisher’s *Z*-transformed, and the overall average was calculated and back-transformed. This was done for the whole group, and separately for smokers and nonsmokers. Unpaired *t* tests on Fisher’s *Z*-transformed correlations were used to determine if correlations for smokers and nonsmokers were significantly different from zero and from each other (*p* ≤ .01). In addition, to examine variability across flavors, a Pearson correlation between smelling and vaping was calculated for each of the 25 e-liquid flavors separately.

Finally, within smelling and vaping data, we calculated Pearson correlation coefficients between the four variables (using ratings across all participants and flavors). *T* tests were used to determine if correlations were significantly different from zero (*p* ≤ .05).

## Results

### Mean Liking, Intensity, Familiarity, Irritation Ratings of E-Liquid Flavors

Mean ratings for liking, familiarity, and irritation showed no significant effects for assessment type, smoking status, and their interaction (see Table 1). A significant interaction term was found for intensity (*p* = .01 after false discovery rate correction). Intensity ratings were higher for smelling compared to vaping (for both user groups), and nonsmokers rated the flavors as more intense compared to smokers (for both assessment types). The significant interaction reflects particularly low intensity ratings for assessment by means of vaping in smokers.

**Table 1.** Group Means (±SE) and Two-Way ANOVA *p* Values for Liking, Intensity, Familiarity, and Irritation Ratings

	Smokers (*n* = 24)		Nonsmokers (*n* = 24)		Two-way ANOVA (FDR corrected *p* values)			
	Smelling	Vaping	Smelling	Vaping	Assessment type	Smoking status	Interaction
Liking	−1.4 ± 1.0	0.8 ± 0.9	1.2 ± 0.9	−0.1 ± 0.9	.39	.17	.17
Intensity	9.6 ± 0.8	3.5 ± 0.8	12.1 ± 0.8	11.4 ± 0.8	.56	.17	.01*
Familiarity	7.0 ± 1.0	2.5 ± 1.1	4.8 ± 1.0	2.1 ± 1.1	.17	.23	.43
Irritation	−22.1 ± 0.9	−26.2 ± 0.8	−23.5 ± 0.8	−25.5 ± 0.8	.17	.27	.27

FDR = false discovery rate. The ANOVA model included assessment type (smelling vs. vaping) and smoking status (smokers vs. nonsmokers). Data were collected on a 0- to 100-mm Visual Analog Scale (anchored “not at all” to “extremely”) and centered around zero by subtracting a constant value of 50.

*Significant (*p* ≤ .05) after FDR correction.

For the individual e-liquid flavors, mean liking ratings did not significantly differ between smokers and nonsmokers. Furthermore, there were no significant differences between smokers and nonsmokers regarding intensity, familiarity, and irritation (except vaping of caramel, which scored significantly higher in intensity for nonsmokers compared to smokers; *p* = .04 after false discovery rate correction). Information on mean ratings for liking, intensity, familiarity, and irritation for smelling and vaping of individual flavors can be found in Supplementary Figures S1–S4.

### Correlations Between Smelling and Vaping

**Figure 1.**
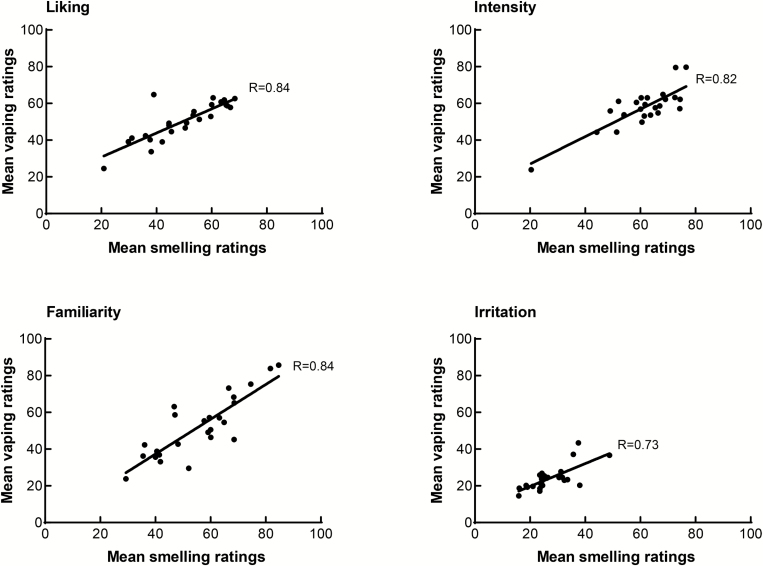
Correlation coefficients between smelling and vaping based on the mean group ratings of each of the 25 products, for liking (top left), intensity (top right), familiarity (bottom left), and irritation (bottom right). Each dot represents the mean group rating for a product on a 100-mm Visual Analog Scale. The same data are presented as mean of within-subject correlation coefficients in Table 2.

The correlation coefficient between smelling and vaping for liking, based on mean group ratings, was 0.84. Figure 1 shows the correlation coefficients based on the mean smelling and vaping ratings of the whole group for all variables. The correlation coefficients separately for smokers and nonsmokers can be found in the Supplementary Materials.

**Table 2.** Mean of the 48 Within-Subjects Correlation Coefficients Between Smelling and Vaping for Liking, Intensity, Familiarity, and Irritation, for the Whole Group (*n* = 48) and Separately for Smokers (*n* = 24) and Nonsmokers (*n* = 24)

	Whole group	Smokers	Nonsmokers	*p* Value*
Liking	0.51	0.49	0.54	.48
Intensity	0.37	0.34	0.40	.28
Familiarity	0.47	0.44	0.50	.31
Irritation	0.25	0.21	0.29	.24

The same data are presented as correlation coefficients based on mean group ratings in Figure 1.

*Correlations between smokers and nonsmokers were considered significantly different if *p* ≤ .01.

The mean of the within-subject correlations between smelling and vaping for liking was 0.51. Table 2 shows the means of the within-subject correlation coefficients for all variables. For each variable, correlations were significantly different from zero (*p* ≤ .01). None of the correlations differed significantly between smokers and nonsmokers. The correlations for liking separated by flavor and participant can be found in the Supplementary Materials.

### Correlations Within Smelling and Vaping

Table 3 shows the correlation coefficients between ratings for liking, intensity, familiarity, and irritation over the whole group and all flavors. For both smelling and vaping, significant correlations were found between liking and familiarity (*R* = 0.45 and *R* = 0.37 for smelling and vaping, respectively), liking and irritation (*R* = −0.29 and *R* = −0.16), intensity and familiarity (*R* = 0.34 and *R* = 0.36), and between intensity and irritation (*R* = 0.35 and *R* = 0.29). In addition, for vaping, significant correlations were found between liking and intensity (*R* = −0.07) and between familiarity and irritation (*R* = 0.08).

**Table 3.** Pearson Correlation Coefficients Between Liking, Intensity, Familiarity, and Irritation, for Smelling and Vaping, for Smokers and Nonsmokers Combined

	Smelling				Vaping			
	Liking	Intensity	Familiarity	Irritation	Liking	Intensity	Familiarity	Irritation
Liking	1.00	−0.04	0.45*	−0.29*	1.00	−0.07*	0.37*	−0.16*
Intensity		1.00	0.34*	0.35*		1.00	0.36*	0.29*
Familiarity			1.00	0.02			1.00	0.08*
Irritation				1.00				1.00

*Significantly different from zero (*p* ≤ .05).

## Discussion

This study aimed to investigate if hedonic evaluation of e-liquid flavors by orthonasal smelling is correlated with (retronasal) vaping ratings. We found strong positive group-level correlations and more modest within-subject correlations between smelling and vaping for ratings of liking, intensity, familiarity, and irritation of e-liquid flavors. Correlations between smelling and vaping varied across individuals and flavors, but did not differ between smokers and nonsmokers.

The strong positive correlations between smelling and vaping are in line with previous studies that found comparable results between orthonasal and retronasal perception of food products.^30–32,40,41^ This can be explained by physiological reasons, as both orthonasal (smelling) and retronasal smell (vaping) cause the volatile flavor molecules to be sensed by the same olfactory receptors located in the nasal epithelium. The strong group-level correlations between smelling and vaping justify the use of orthonasal smelling (instead of vaping) e-liquids to measure hedonic flavor perception in studies where vaping would be inappropriate or not feasible. Examples of such research situations are investigating nicotine-naïve individuals (ie, nonusers) or individuals under legal age for e-cigarette use (ie, youth and adolescents). In addition, smelling can be used to compare hedonic flavor assessment between adolescent nonusers and current smokers, providing support for future regulations on e-liquid flavors. Finally, neural responses to e-liquid flavor/odor exposure (eg, using functional magnetic resonance imaging or electroencephalogram) can help to better understand the role of flavors in liking of and reward from e-cigarettes.

This study showed that the correlations between smelling and vaping varied across flavors. The correlation between smelling and vaping for liking of whiskey flavor was negative, potentially because the whiskey-flavored e-liquid received the lowest ratings for liking. The positive correlations for other flavors varied from modest to strong (see Supplementary Material). As we used only one or two e-liquids to represent a main flavor category, the across-flavors variability in correlation coefficients could be assigned to the individual products selected rather than to flavor categories in general. Consequently, smelling experiments can be used in the future to investigate overall flavor liking among different user groups. However, in order to investigate differences between flavor categories or even between individual flavors, each category or individual flavor should be represented by multiple e-liquids from various subcategories (eg, e-liquids with a mojito, beer, and rum flavor to represent the “alcohol” category) or brands (eg, multiple strawberry- or orange-flavored e-liquids from different brands).

### Correlations Within Smelling and Vaping

The correlations within smelling and vaping showed that liking was positively correlated with familiarity and negatively with irritation, which is in line with previous literature.^28,35^ There was no correlation for smelling between liking and intensity, and a low negative correlation for vaping (ie, the higher the intensity ratings, the less a flavor was liked). This could be explained by the typically nonlinear, inverted “U” shaped relation between intensity and liking, where liking first increases with physical (or sensory) intensity, peaks, and then decreases.^42^ Since commercial e-liquids were used, it may be assumed that the flavors were designed to have an intensity that results in optimal liking ratings (peak of the curve). Following the inverted “U” shaped curve, higher intensity ratings would then indeed result in lower ratings for liking. This is supported by, for instance, outcomes for the strawberry-flavored e-liquid: for smelling, mean intensity ratings were much higher and, consequently, mean liking ratings were much lower than for vaping.

In addition, this study found higher intensity ratings for smelling than for vaping. In line with this, previous research showed that odors presented in an orthonasal way were rated as more intense than odors administered in a retronasal way.^40,41^ An explanation may be that odors are typically encountered at higher concentrations during orthonasal perception of liquids versus retronasal perception.^40,43^ Therefore, it should be taken into account that orthonasal compared to retronasal presentation of e-liquid flavors may require lower concentrations to produce the same intensity when replacing future vaping experiments by smelling experiments. Future research is needed to determine an optimal and consistent e-liquid intensity for conducting smelling experiments. A possible approach may be heating the e-liquids, as increasing temperature may change flavor perception due to an increased release of volatile molecules.^44,45^

### Comparing Smokers and Nonsmokers

This study found that smokers perceived the flavors as less intense than nonsmokers did; intensity ratings were particularly low for assessment by means of vaping in smokers. Although smokers are more prone to olfactory dysfunction than nonsmokers,^46^ we only included participants with normal olfactory function in our study. However, smokers may have rated intensity during vaping lower because they are used to inhale smoke.

In addition, we found that the flavors rated highest and lowest in liking differed between smokers and nonsmokers (see Supplementary Material). Whereas liking was highest for mint and peppermint among smokers, sweet flavors such as strawberry, watermelon, and caramel scored highest for liking among nonsmokers. Although this may suggest a trend that is in line with previous literature,^12^ differences in flavor liking between smokers and nonsmokers were not significant. A reason for this may be that we asked participants to focus on flavor perception only (*how much do you like this flavor?*) rather than creating an e-cigarette context (eg, *how much would you like to try an e-cigarette with this flavor?*). Because liking depends on context factors^42^ and flavor preference in an e-cigarette context may differ between user groups,^8,9,20–22^ differences in hedonic flavor assessment between user groups may have been found if questions were to be asked in an e-cigarette context. In addition, as people are often unable to identify unlabeled flavors without a predefined list of verbal descriptors to choose from^47^ and learned associations from previous experiences can influence the hedonic perception,^48^ outcomes may have been different if participants would be aware of the specific flavors used in this study. Overall, our study design was chosen to determine the correlation between smelling and vaping for liking of e-liquid flavors. Future research investigating differences in flavor liking between user groups, for example, using smelling experiments, would benefit from creating an e-cigarette context and labeling the flavors under investigation.

### Strengths of This Study

A strength of this study was that we included participants who had never used an e-cigarette; thus, outcomes were not influenced by prior vaping experiences. In addition, the panel consisted of a balanced number of smokers and nonsmokers (50% were smokers), and both user groups had a similar mean age and equal male/female ratio (50% were male). Finally, we selected e-liquid flavors from all different flavor categories^37^ and covered a wide hedonic range in order to rule out strong influences from individual flavors on the overall correlations.

### Limitations and Future Directions

The more modest within-subject correlations, variation across individuals, and variation across specific e-liquid flavors found in this study suggest that future research is needed to investigate whether the use of smelling instead of vaping is applicable to other research situations. First, this study used nicotine-free e-liquids (for ethical reasons), but the use of nicotine-containing e-liquids may have resulted in different outcomes. That is, nicotine may be expected to evoke taste and chemesthetic sensations during vaping (ie, bitterness and harshness) that contribute to flavor (dis)liking. As these sensations cannot be perceived by means of orthonasal smelling, a research situation that includes nicotine-containing e-liquids may yield lower correlations between hedonic smelling and vaping ratings. In addition, nicotine may cause participants to have more difficulties restricting their ratings to odor/flavor perception without being influenced by the overall vaping experience. Future studies are thus necessary to determine the degree to which smelling and vaping ratings align when using nicotine-containing e-liquids.

Second, even though previous literature showed that e-liquid flavor perception more strongly depends on (retronasal) olfaction than on taste,^33^ it would be interesting to investigate the role of taste in orthonasal assessment of e-liquid flavors (eg, via learned associations). Additionally, it should be noted that e-liquids with an identical flavor label (eg, melon from brand A and melon from brand B) might not consist of the same mixture of odor molecules and thus differ in the response pattern in the olfactory epithelium and beyond.^49^ Hence, while our results justify orthonasal assessment of affective responses, additional research is needed to determine whether orthonasal smelling can also be used for assessments of sensory (perceptual) responses such as descriptive odor profiles of e-liquids. Finally, as smelling experiments were previously used to identify characterizing flavors in cigarettes and roll-your-own tobacco,^50^ future research could focus on expanding the current results to flavors in other product types such as water pipe, cigars, and heated tobacco products.

### Concluding Remarks

We are the first to show that hedonic evaluation through orthonasal (smelling) and retronasal assessment (vaping) of e-liquid flavors yields comparable results, for both smokers and nonsmokers. This finding justifies the use of orthonasal smelling instead of vaping in several future studies, for example, investigating individuals who are nicotine-naïve (ie, nonusers) or under legal age for e-cigarette use (ie, adolescents). Thus, smelling experiments, also being faster and less expensive than vaping, might be used to provide support for future regulations on e-liquid flavors. However, the more modest within-subject correlations and variation across individuals and specific e-liquid flavors suggest that the use of smelling instead of vaping may not be applicable to all research situations (eg, for nicotine-containing e-liquids). Additional research is necessary to understand which variables tend to dissociate smelling versus vaping ratings.

## Supplementary Material

ntz155_suppl_Supplementary_Information
