# Supplementary material for: Sensory Evaluation of E-Liquid Flavors by Smelling and Vaping Yields Similar Results
Source: Nicotine Tob Res. 2019 Aug 22;22(5):798–805. doi: 10.1093/ntr/ntz155 (PMC7171284; doi:10.1093/ntr/ntz155)
Supplement: ntz155_suppl_Supplementary_Information [file ntz155_suppl_supplementary_information.pdf]

Sensory evaluation of e-liquid flavors by smelling and vaping yields similar results

Erna JZ Krüsemann, MSc. [1,2*]; Franziska M Wenng[2]; Jeroen LA Pennings, PhD [1]; Kees de Graaf, Prof [2]; Reinskje Talhout, PhD [1]; Sanne Boesveldt, PhD [2]

Institutional addresses:

[1] Centre for Health Protection, National Institute for Public Health and the Environment (RIVM), Antonie van Leeuwenhoeklaan 9, 3721 MA Bilthoven, The Netherlands

[2] Division of Human Nutrition and Health, Wageningen University, Stippeneng 4, 6708 WE Wageningen, The Netherlands

* Corresponding author: erna.krusemann@rivm.nl

Table of Contents

Panel characteristics 2

Mean liking, intensity, familiarity, irritation ratings of e-liquid flavors 3

Mean liking ratings for smokers and non-smokers 8

Correlations between smelling and vaping among smokers and non-smokers 10

Correlations between smelling and vaping for liking 10

Panel characteristics

Table S1: Panel characteristics

		Whole group (n=48)	Smokers (n=24)	Non-smokers (n=24)*
**Gender**	Female	50%	50%	50%
	Male	50%	50%	50%
**Mean age (± SD)**		24.8±8.4	24.8±9.3	24.9±7.7
**Age range**		18-55	18-54	20-55
**Mean number of cigarettes/day (± SD)**		NA	10.2±6.5	NA
**Participants who inhaled during vaping session via****				
	mouth only	19	1	18
	mouth and lungs	28	23	5
	don't remember	1	0	1
**Participants who exhaled during vaping session via****				
	mouth	36	16	20
	mouth and nose	12	8	4

* Among the group of non-smokers, there were 2 ex-smokers who reported to have quit smoking for 6 and7 years, respectively.

** Inhalation and exhalation approach were asked retrospectively.

Mean liking, intensity, familiarity, irritation ratings of e-liquid flavors

Mean liking ratings ranged from 20.9±2.0 (whiskey) to 68.4±2.1 (mint) for smelling, and from 24.5±2.8 (whiskey) to 64.8±2.3 (strawberry) for vaping (see Figure S1). Intensity ratings ranged from 20.4±2.7 (PG/VG base) to 76.6±2.2 (mint) for smelling, and from 23.9±2.5 (PG/VG base) to 79.7±1.6 (mint) for vaping (see Figure S2). Familiarity ranged from 29.3±3.0 (PG/VG base) to 84.7±1.4 (mint) for smelling, and from 23.8±2.3 (PG/VG base) to 85.7±1.2 (mint) for vaping (see Figure S3). Irritation ranged from 15.9±2.3 (PG/VG base) to 48.8±3.7 (whiskey) for smelling, and from 14.5±1.6 (PG/VG base) to 43.4±3.7 (peppermint) for vaping (see Figure S4).

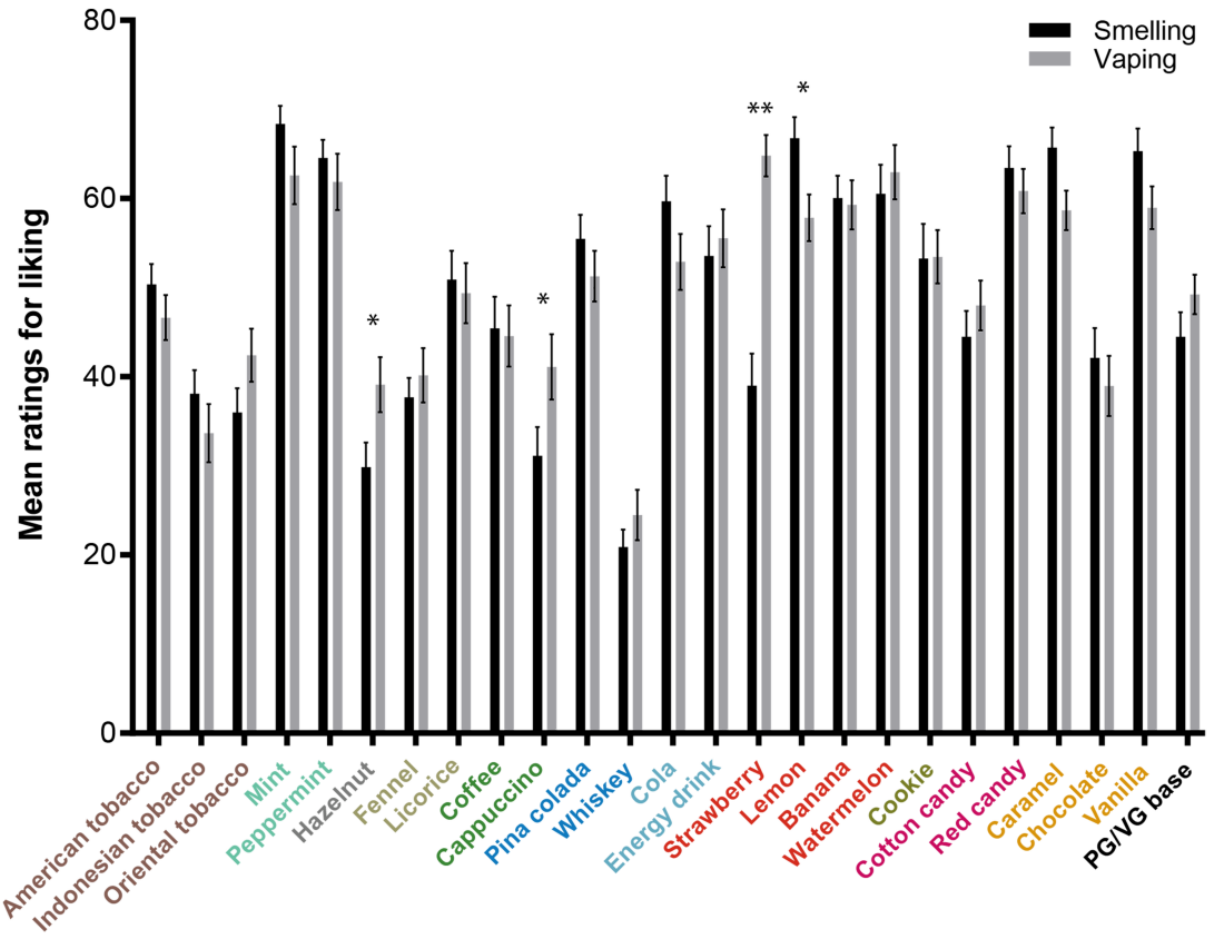

Figure S1: Mean ratings (±SE) for **liking** of each e-liquid flavor based on results from the whole group on a 100 mm VAS, for smelling (black) and vaping (grey). * significant difference between smelling and vaping with $p < 0.05$ (after false discovery rate correction); ** significant difference between smelling and vaping with $p < 0.001$ (after false discovery rate correction)

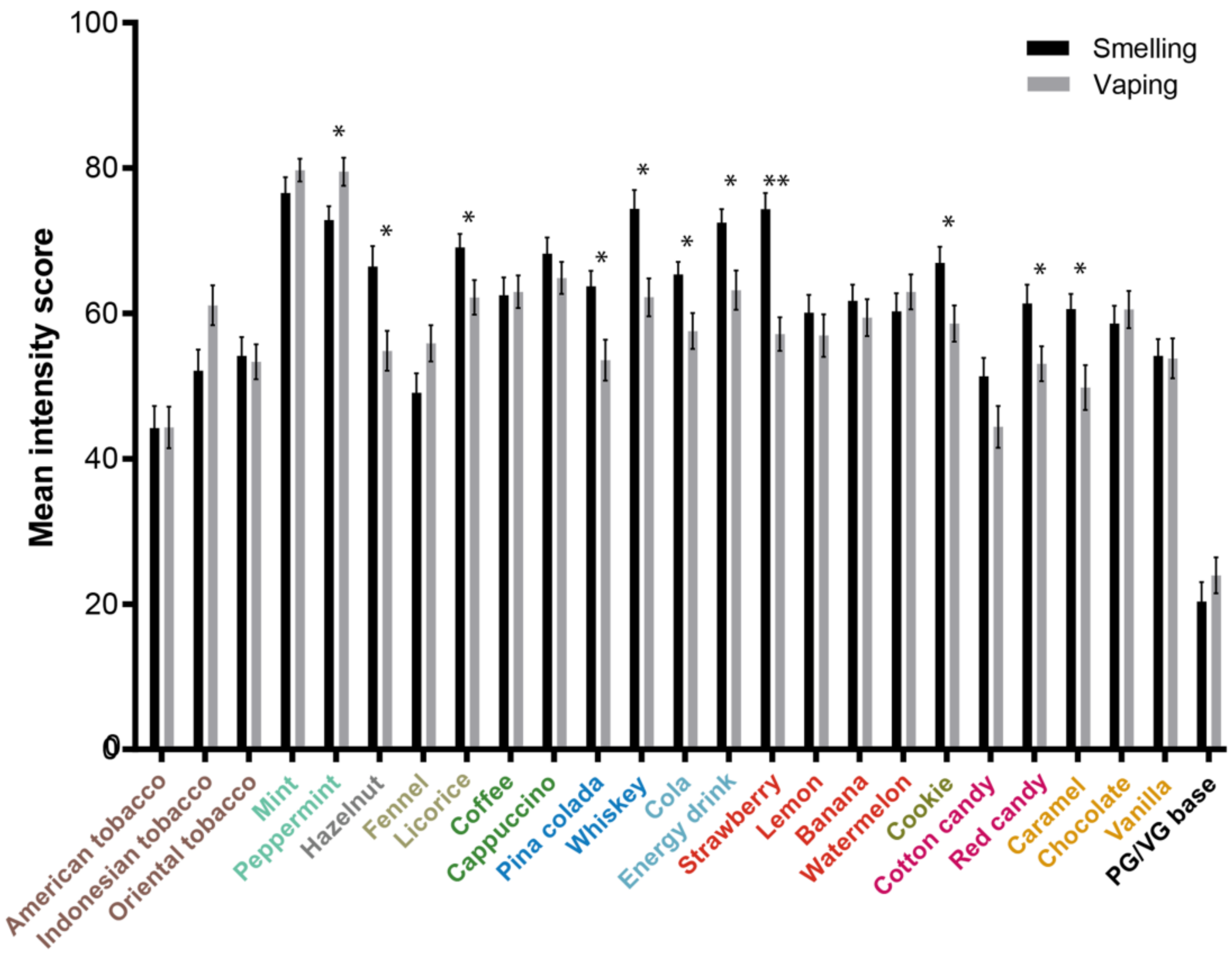

Figure S2: Mean ratings (±SE) for **intensity** of each e-liquid flavor based on results from the whole group on a 100 mm VAS, for smelling (black) and vaping (grey). * significant difference between smelling and vaping with $p < 0.05$ (after false discovery rate correction); ** significant difference between smelling and vaping with $p < 0.001$ (after false discovery rate correction)

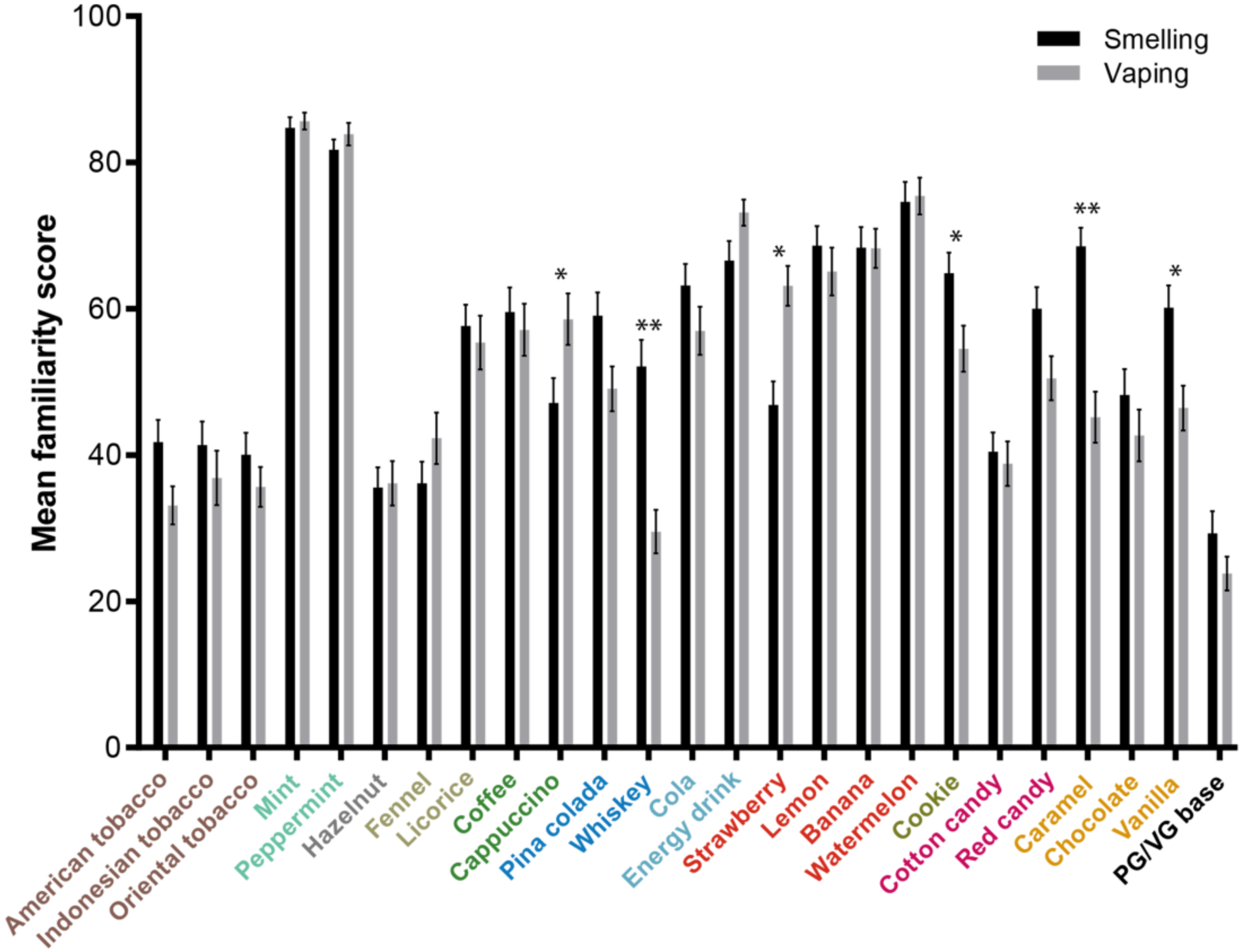

Figure S3: Mean ratings (±SE) for **familiarity** of each e-liquid flavor based on results from the whole group on a 100 mm VAS, for smelling (black) and vaping (grey). * significant difference between smelling and vaping with $p < 0.05$ (after false discovery rate correction); ** significant difference between smelling and vaping with $p < 0.001$ (after false discovery rate correction)

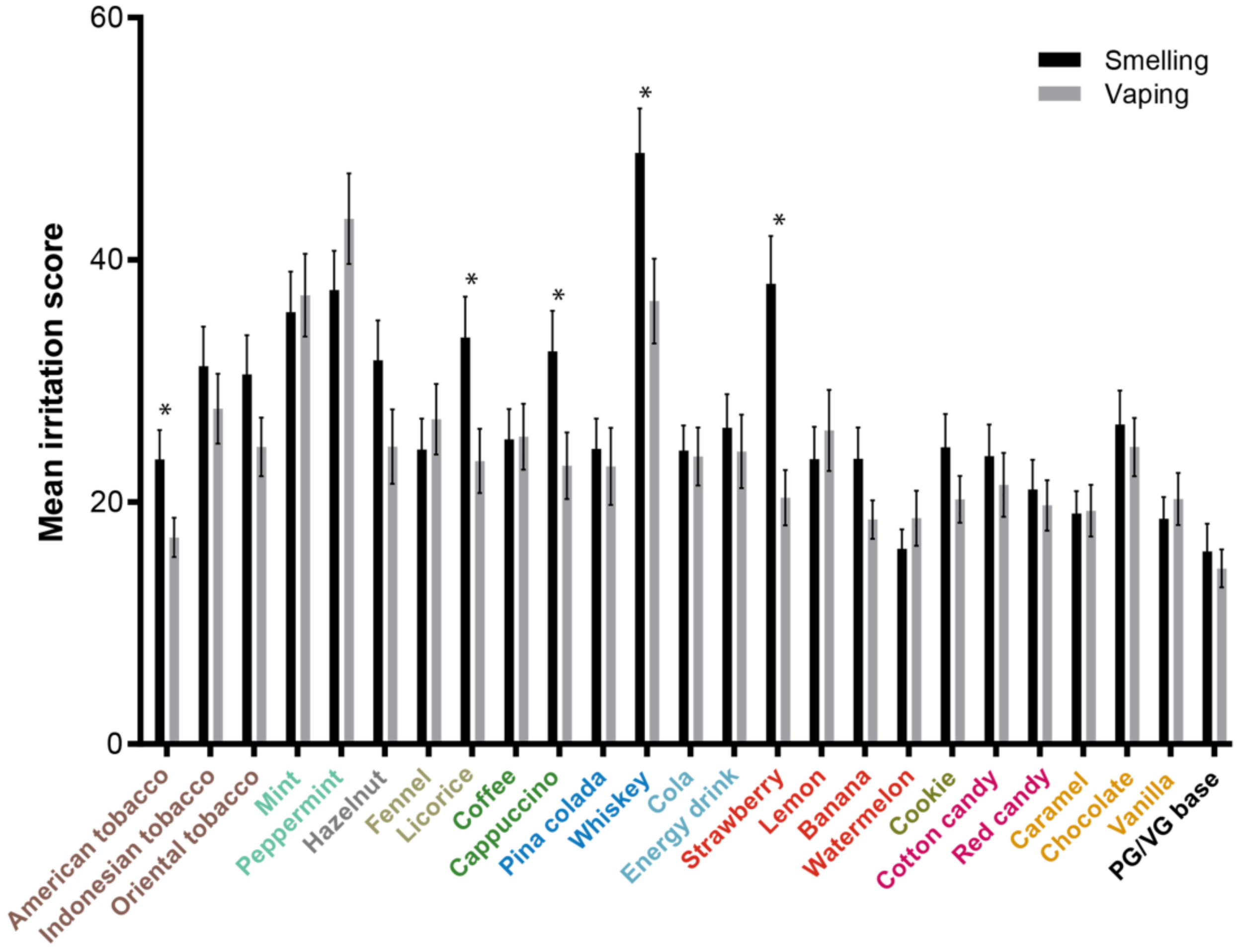

Figure S4: Mean ratings (±SE) for **irritation** of each e-liquid flavor based on results from the whole group on a 100 mm VAS, for smelling (black) and vaping (grey). * significant difference between smelling and vaping with $p < 0.05$ (after false discovery rate correction); ** significant difference between smelling and vaping with $p < 0.001$ (after false discovery rate correction)

Mean liking ratings for smokers and non-smokers

Mean liking ratings among smokers was higmhest for mint (69.9±3.0) followed by peppermint (67.8±2.6) in smelling, and for peppermint (68.5±4.2) and mint (65.4±4.9) in vaping (see Table S2). Among non-smokers, mean liking was highest for lemon (67.9±3.3) and caramel (67.2±2.6) in smelling, and for strawberry (66.0±3.2) and watermelon (64.2±3.7) in vaping. Mean liking among smokers was lowest for whiskey (19.9±3.0) and hazelnut (27.3±4.5) in smelling, and whiskey (26.7±4.7) and Indonesian tobacco (32.4±4.7) in vaping. Among non-smokers, mean liking was lowest for whiskey (21.9±2.5) and cappuccino (30.8±4.4) in smelling, and for whiskey (22.3±3.2) and Indonesian tobacco (34.9±4.5) in vaping. However, differences in mean flavor liking between smokers and non-smokers were not significant. See Table S1 for a complete overview of the mean ratings for liking from both user groups.

Table S2: Mean liking ratings for smokers and non-smokers. Data were collected on a 0 to 100 mm VAS. Within each group, the two highest ratings are marked in green and the two lowest ratings are marked in red. Differences in mean flavor liking between smokers and non-smokers were not significant (p>0.05).

	**Whole group**		**Smokers**		**Non-smokers**	
	Smelling	Vaping	Smelling	Vaping	Smelling	Vaping
**American tobacco**	50.4 ± 2.3	46.6 ± 2.5	49.1 ± 2.9	48.8 ± 3.8	51.7 ± 3.5	44.5 ± 3.3
**Banana**	60.0 ± 2.5	59.3 ± 2.8	58.6 ± 3.9	59.3 ± 4.5	61.5 ± 3.3	59.3 ± 3.3
**Cappuccino**	31.1 ± 3.2	41.1 ± 3.7	31.5 ± 4.8	41.3 ± 5.1	30.8 ± 4.4	40.9 ± 5.3
**Caramel**	65.7 ± 2.3	58.7 ± 2.2	64.2 ± 3.7	56.7 ± 3.4	67.2 ± 2.6	60.6 ± 2.8
**Chocolate**	42.1 ± 3.4	39.0 ± 3.4	44.9 ± 5.0	37.9 ± 4.9	39.4 ± 4.5	40.1 ± 4.7
**Coffee**	45.4 ± 3.6	44.6 ± 3.4	48.0 ± 5.2	48.9 ± 5.0	42.9 ± 5.0	40.2 ± 4.6
**Cola**	59.7 ± 2.9	52.9 ± 3.1	60.8 ± 4.0	54.0 ± 3.7	58.6 ± 4.2	51.8 ± 5.1
**Cookie**	53.3 ± 3.9	53.5 ± 3.0	48.2 ± 6.2	49.9 ± 4.4	58.3 ± 4.6	57.1 ± 4.0
**Cotton candy**	44.5 ± 2.9	48.0 ± 2.8	39.9 ± 4.5	48.8 ± 4.1	49.1 ± 3.4	47.2 ± 4.0
**Energy drink**	53.6 ± 3.3	55.5 ± 3.2	48.9 ± 4.9	54.5 ± 5.3	58.2 ± 4.4	56.6 ± 3.8
**Fennel**	37.7 ± 2.2	40.2 ± 3.1	35.9 ± 3.2	39.4 ± 4.1	39.5 ± 3.0	40.9 ± 4.6
**Hazelnut**	29.8 ± 2.8	39.1 ± 3.1	27.3 ± 4.5	39.3 ± 4.6	32.4 ± 3.3	38.9 ± 4.2
**Indonesian tobacco**	38.1 ± 2.7	33.7 ± 3.3	37.3 ± 3.7	32.4 ± 4.7	39.0 ± 3.9	34.9 ± 4.5
**Lemon**	66.8 ± 2.4	57.8 ± 2.6	65.6 ± 3.4	53.2 ± 4.4	67.9 ± 3.3	62.5 ± 2.6
**Licorice**	50.9 ± 3.2	49.4 ± 3.4	46.3 ± 5.0	48.7 ± 4.9	55.5 ± 4.0	50.1 ± 4.8
**Mint**	68.4 ± 2.1	62.6 ± 3.2	69.9 ± 3.0	65.4 ± 4.9	66.8 ± 2.8	59.8 ± 4.2
**Oriental tobacco**	36.0 ± 2.7	42.4 ± 3.0	34.3 ± 4.3	47.3 ± 4.8	37.7 ± 3.4	37.5 ± 3.4
**Peppermint**	64.6 ± 2.0	61.9 ± 3.1	67.8 ± 2.6	68.5 ± 4.2	61.4 ± 3.0	55.3 ± 4.4
**PG/VG Base**	44.5 ± 2.8	49.2 ± 2.2	42.2 ± 3.5	46.5 ± 3.5	46.8 ± 4.2	52.0 ± 2.7
**Piña colada**	55.5 ± 2.7	51.3 ± 2.9	57.1 ± 4.0	55.9 ± 4.2	53.9 ± 3.6	46.7 ± 3.7
**Red candy**	63.4 ± 2.5	60.8 ± 2.5	61.0 ± 3.6	62.4 ± 3.2	65.9 ± 3.4	59.3 ± 3.8
**Strawberry**	39.0 ± 3.6	64.8 ± 2.3	33.5 ± 5.1	63.6 ± 3.4	44.5 ± 4.9	66.0 ± 3.2
**Vanilla**	65.3 ± 2.6	59.0 ± 2.4	65.2 ± 3.4	59.4 ± 2.5	65.4 ± 3.9	58.5 ± 4.2
**Watermelon**	60.5 ± 3.3	63.0 ± 3.1	57.7 ± 4.9	61.7 ± 4.9	63.3 ± 4.4	64.2 ± 3.7
**Whiskey**	20.9 ± 2.0	24.5 ± 2.8	19.9 ± 3.0	26.7 ± 4.7	21.9 ± 2.5	22.3 ± 3.2

Correlations between smelling and vaping among smokers and non-smokers

For smokers, the correlation coefficients based on the mean smelling and vaping ratings were 0.78 for liking, 0.76 for intensity, 0.83 for familiarity, and 0.65 for irritation. For non-smokers, the correlation coefficients based on the mean smelling and vaping ratings were 0.86 for liking, 0.85 for intensity, 0.84 for familiarity, and 0.75 for irritation. The correlations did not significantly differ between smokers and non-smokers.

Correlations between smelling and vaping for liking

The correlations for liking between smelling and vaping were positive for all individuals, except for two subjects (R=-0.27 and R=-0.06). The positive correlation coefficients ranged from 0.06 to 0.87. The correlation coefficients for liking of the individual e-liquid flavors between smelling and vaping (n=25) were all positive, except for whiskey (R=-0.33). The positive correlations ranged between 0.08 (caramel) and 0.81 (watermelon).
